# Aloe-Emodin Ameliorates Diabetic Nephropathy by Targeting Interferon Regulatory Factor 4

**DOI:** 10.1155/2022/2421624

**Published:** 2022-04-26

**Authors:** Ling Lu, Yin Li

**Affiliations:** ^1^Department of Nephrology, Tianjin First Central Hospital, School of Medicine, Tianjin, China; ^2^Department of Intensive Care Unit, Tianjin First Central Hospital, School of Medicine, Tianjin, China

## Abstract

Diabetic nephropathy (DN) is one of the leading causes of end-stage renal disease and lacks effective clinical treatment for its complicated pathogenesis. In this study, the gene expression profiles downloaded from the GEO database were used to identify the key regulatory gene through bioinformatics analyses, and the potential mechanism in regulating DN was revealed via the gene set enrichment analysis, pathway analysis, and in vitro phenotype detection. The effect of the screened drug on DN was analyzed through in vitro and in vivo model experiments. Interferon regulatory factor 4 (IRF4) in DN was identified to be upregulated compared with that in normal control tissues. Further results revealed that IRF4 promoted the DN progression through inflammation, immunity, and extracellular matrix remodeling. The screening results of the TCM library showed that aloe-emodin (Ae) should be a potentially active target drug, and the in vitro and in vivo experiment results demonstrated that Ae could ameliorate DN by targeting IRF4. In conclusion, this study revealed the mechanism of the DN progression and demonstrated that Ae could be a potential target drug in ameliorating DN, providing ideas for the clinical treatments for DN.

## 1. Introduction

Diabetic nephropathy (DN) is one of the main complications of diabetes [1–3]. DN is the main cause of end-stage renal failure [4]. The pathogenesis of DN is complex, and the exact mechanism remains unclear [5]. At present, hemodynamic changes, metabolic disorders, inflammation, autophagy, oxidative stress, miRNAs, and some pathological factors are believed to be involved in the occurrence and the development of the disease [6]. Although strictly controlling the blood sugar and the blood pressure can delay the progression of DN, an ideal intervention to reverse or prevent the progression of DN remains unavailable [7].

The interferon regulatory factor (IRF) is a transcription factor that acts on many biological processes [8]. IRF is involved in immune regulation by regulating the interferon expression, differentiation, development, and activity of hematopoietic cells [9]. IRF can also be involved in the occurrence of tumors by regulating cell proliferation and apoptosis. The role of different IRFs in immune regulation is evidently different. The IRF4 is a member of the IRF family [8, 10]. However, few studies have reported whether IRF4 is involved in the occurrence and the development of DN.

Aloe-emodin (Ae) is an anthraquinone found in aloe, rhubarb, cassia seed, and other Chinese herbs [11]. Ae has a chemical name of 1,8-dihydroxy-3-hydroxymethyl-9,10-anthraquinone, molecular formula of C_15_H_10_O_5_, and relative molecular weight of 270.23 [12]. The pharmacological action is closely related to the chemical structure. The anthraquinone ring and two phenolic hydroxyl groups in Ae determine its scavenging oxygen free radicals and antitumor biological activities [13–15]. Although some reports had demonstrated that the Chinese herbs of rhubarb, aloe vera, and cassia or Ae analogues such as emodin had anti-DN effects [16–19], whether Ae has effects on treatment with DN and the mechanism remain unavailable.

In this study, IRF4 was identified to be a potential target of Ae through drug screening; however, the role of IRF4 in the DN remains unclear, and the mechanism of Ae in the treatment of DN has not been fully understood. On the basis of the bioinformatics analysis and in vitro and in vivo models of DN, the protective effect of the IRF4 knockdown on DN is studied. Moreover, considering IRF4 and its downstream pathway, the protective effect and the mechanism of Ae on DN in rats are further studied.

## 2. Materials and Methods

### 2.1. Omics Analysis

The gene expression profiles of GSE142025 downloaded from the GEO database were analyzed in this study. A total of 21 kidney samples from patients with DN and 9 control human kidney samples were included in this database. Differentially expressed genes were analyzed using the R packages of pheatmap and limma among these samples. The protein-protein interaction (PPI) annotation was achieved using the STRING database and the Cytoscape app. The R packages of the clusterProfiler and the ggplot2 were used for the gene set enrichment analysis (GSEA) and the pathway analyses.

### 2.2. Cell Culture and Transfection

The immortalized human podocytes AB8/13 purchased from the American Type Culture Collection were cultured in the RPMI 1640 medium (Hyclone, USA) containing 5.5 mM glucose with 10% fetal bovine serum (Hyclone, USA). AB8/13 cells were cultured at 33°C in 5% CO_2_ atmosphere with the addition of insulin-transferrin-selenium (Life Technologies) and at 37°C in 5% CO_2_ atmosphere without insulin-transferrin-selenium for more than seven days to induce their differentiation [20]. The differentiated podocytes were cultured with RPMI 1640 media containing 5.5 (control group) or 30 (model group) mM glucose for 24 h [21], the differentiated podocytes in the Ae group were treated with RPMI 1640 media containing 30 mM glucose and 2 *μ*M Ae for 24 h. The shRNA-IRF4 plasmid obtained from the GeneCopoeia (Guangzhou, China) was transfected to model cells with transfection reagents (Roche, Switzerland) in accordance with the manufacturer's instructions. 5 × 10^5^ cells/well were seeded into the 6-well culture plates, when cells were attached to the plate, the RNA-lipid complexes containing 1 *μ*g of shRNA-IRF4 plasmid and 3 *μ*L transfection reagent in 100 *μ*L Opti-MEM medium were added to the cell culture per well for 24 h.

### 2.3. Western Blot Analysis

The protein (30 *μ*g) collected by RIPA lysate buffer per group was used for the western blot. The primary antibodies of IRF4, collagen I, Notch 1, p-AKT, AKT, and GAPDH (CST, USA) and secondary antibodies (CST, USA) were used in this experiment. Blots were visualized with the enhanced chemiluminescence detection kit (Millipore, USA). The densitometric analysis was accomplished using the ImageJ software. Each experiment was repeated in triplicate, and mean values (mean ± s.d.) were presented.

### 2.4. Enzyme-Linked Immunosorbent Assay (ELISA)

Commercial ELISA kits (Abcam, USA) for IL-1*β* and IL-7 were used to measure the factor concentrations in the culture medium or sera in accordance with the manufacturer's instructions. Medium samples from different treatment groups were collected at 48 h. Factor contents were detected using the Luminoskan Ascent Reader System (Thermo Fisher Scientific, USA). Each experiment was repeated in triplicate, and mean values (mean ± s.d.) were presented.

### 2.5. TCM Database and Molecular Docking Simulation of IRF4

A total of 18436 TCM molecules were obtained from the TCM database. All TCM molecules were refined by removing the counterions and salt and adding hydrogen atoms. The energy minimization was performed using the Schrodinger software. For the protein preparation, the crystal structure of the IRF4 was downloaded from the protein data bank. The protein structure was refined by removing crystalline water and ions. Then, hydrogen atoms were added, and the energy minimization of the protein structure was performed. The high-throughput virtual (HTV) screening model of the Schrodinger software was used to perform molecular docking. The Glide XP (extra precision) was used for the final TCM molecule calculation.

### 2.6. Cell Viability Assay

Cell viability was performed using the MTT assay. The differentiated podocytes AB8/13 (5 × 10^3^ cells/mL) were seeded into a 96-well culture plate. Cells were attached to the plate, treated with various concentrations of Ae, and incubated at 37°C and 5% CO_2_ atmosphere for 48 h. The cell viability was measured after adding 20 *μ*L MTT at 37°C for 4 h. Afterwards, 150 *μ*L dimethyl sulfoxide was added to dissolve the formazan crystals. The density was measured at 570 nm by using a microplate reader (Thermo Fisher Scientific, USA).

### 2.7. Animal Studies

The animal study was approved by the Experimental Animal Ethical Committee of Tianjin First Central Hospital and performed in accordance with the National Institutes of Health Guide for the Care and Use of Laboratory Animals. Eighteen Sprague–Dawley rats weighing 250 ± 20 g (equal number of males and females) were divided randomly into three groups after a week of adaptive feeding. Rats in the model (M group) and the model + Ae (20 mg/kg/day, M + Ae group) [22] groups were intraperitoneally injected with 65 mg/kg streptozotocin (STZ, Sigma, USA) dissolved in a 0.1 mM chilled citrate-phosphate buffer to induce diabetes. The rats in the normal control group were injected with the same amount of citrate-phosphate buffer. When the fasting blood glucose levels collected from the tail vein of STZ-induced rats were higher than 16.7 mM at five days after injection, the rats were considered diabetic. A week later, the rats in the M + Ae group were orally treated with Ae dissolved in 0.5% sodium carboxymethyl cellulose at a dose of 20 mg/kg every day. The rats in the control and the M groups were orally treated with the same volume of 0.5% sodium carboxymethyl cellulose. At eight weeks after Ae treatment, the mice were placed in individual metabolic cages to collect urine samples for 24 h and determine the urinary albumin (ALB) level, urinary creatinine (Ucr) level, and ALB level/Ucr level (ACR). Blood samples were collected, and the serum was separated by centrifugation and stored at −80°C until analysis. Right kidney samples were rapidly excised and stored at −80°C until analysis.

### 2.8. Statistical Analysis

All data were presented as the mean ± s.d. After testing for normality and equal variance across the groups, differences among groups were assessed via the Student's *t*-test. All experiments were repeated at least thrice. All data were evaluated using the IBM SPSS software version 22.0 (Chicago, IL, USA). The level of significance was set at *P* < 0.05.

## 3. Results

### 3.1. Selection of the Candidate Key Gene in Regulating the DN Progression

**Figure 1:**
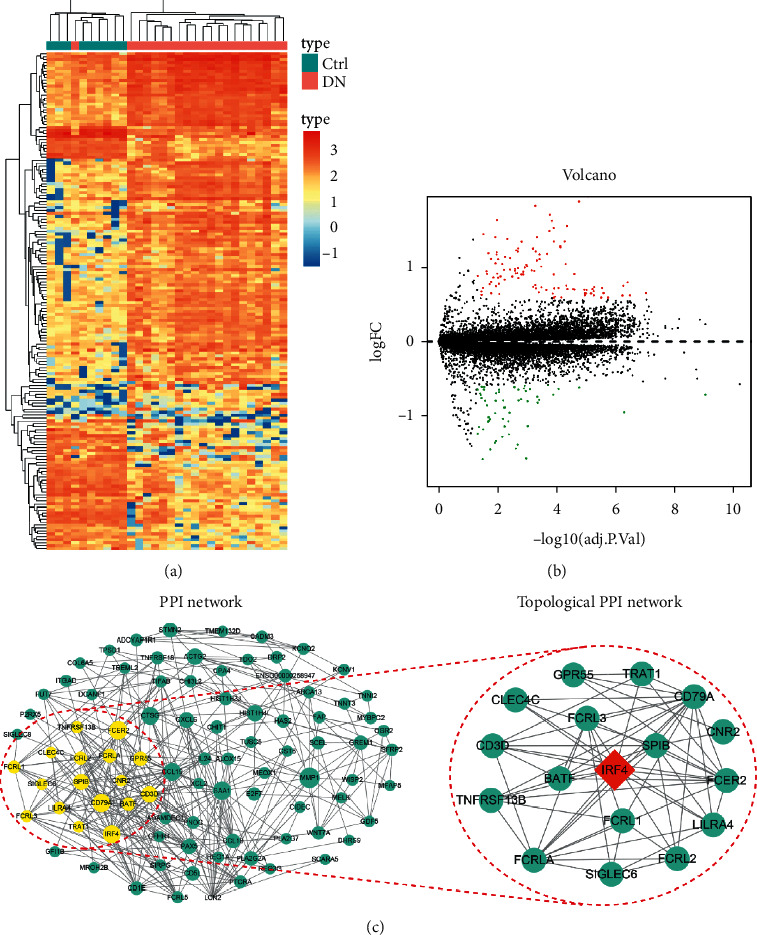
Upregulation of IRF4 in the specimens of patients with DN. (a) Heatmap analysis for the comparison of mRNA expression profiles between normal control and DN samples. (b) Volcano plot for differentially expressed mRNAs between normal control and DN samples. Red and green plots indicate upregulated and downregulated miRNAs, respectively. (c) DN-related PPI hubs as revealed by the PPI network analysis of the upregulated mRNAs. The topological PPI network indicates that IRF4 is a core node gene in regulating DN. DN, diabetic nephropathy; PPI, protein-protein interaction.

The mRNA expression profile data downloaded from the GEO database were first analyzed to screen the candidate key gene involved in the DN progression. The mRNA expression profiles between the control and the DN groups were quite different (Figure 1(a)). Volcano plots further demonstrated that the mRNAs between these two groups were differentially expressed. A total of 111 mRNAs were identified to be upregulated (Figure 1(b)) and subjected to PPI analysis to further screen the core candidate gene. The constructed PPI network consisted of 89 nodes and 378 edges (Figure 1(c), left). The topological PPI network was further screened using the Cytoscape app through the features of Degree and K-core, and the identified topological core PPI network had an MCODE score of 7.625. Finally, the IRF4 gene was identified as the candidate hub core gene in regulating the DN progression (Figure 1(c), right).

IRF4 regulates the DN progression through inflammation, immunity, and extracellular matrix reconstruction.

**Figure 2:**
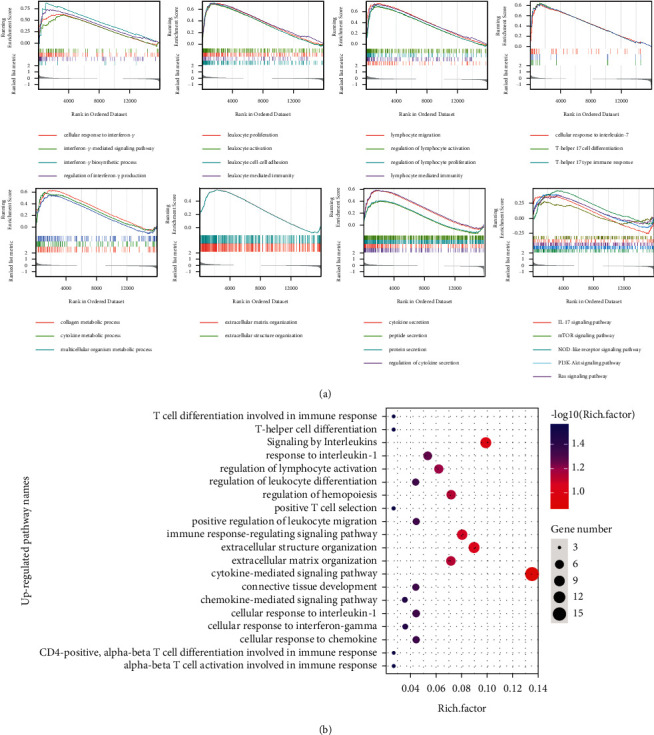
Function and pathway analyses of mRNA expression profiles. (a) Regulations among inflammation, immunity, and extracellular matrix reconstruction as shown by the GSEA results of total mRNA expression profiles. (b) GO and KEGG analyses of the upregulated mRNAs in DN. GSEA, gene set enrichment analysis; GO, gene ontology; KEGG, Kyoto Encyclopedia of Genes and Genomes.

The mRNA expression profile data were first subjected to GSEA to further reveal the mechanism of IRF4 in regulating the DN progression. GSEA results showed that when DN occurred, the pathways and functions related to immunity and inflammation were widely activated. The extracellular structure and matrix organizations were also activated, and these effects led to the reconstruction of the extracellular matrix (Figure 2(a)). Subsequently, unregulated genes were used to perform the Gene Ontology and Kyoto Encyclopedia of Genes and Genomes analyses. Consistent with the GSEA results, analytical results demonstrated that the upregulated genes were widely involved in pathways related to inflammation, immunity, and extracellular matrix reconstruction (Figure 2(b)).

**Figure 3:**
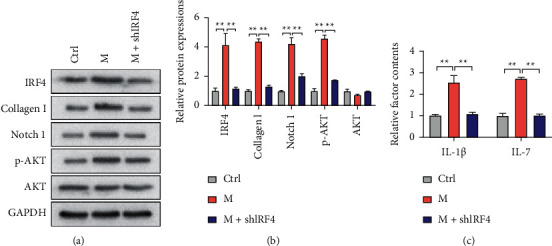
Promotion of the progression of DN by IRF4. (a). Representative bands of IRF4, collagen I Notch 1, p-AKT, AKT, and GAPDH proteins examined by western blot analysis among control, DN model, and DN model + shIRF4-treated AB8/13 cells. (b). Comparison of the gray values of IRF4, collagen I Notch 1, p-AKT, and AKT proteins. (c) ELISA results for the detection of IL-1*β* and IL-7 in cell medium among control, DN model, and DN model + shIRF4-treated AB8/13 cells (mean ± s.d., *n* = 3 in triplicate, ^*∗∗*^*P* < 0.01).

Considering that IRF4 might be the core gene regulating the DN progression and that IRF4 might activate inflammation, immunity, and extracellular matrix reconstruction, in vitro molecular detection experiments were designed to confirm the hypothesis. Western blot results showed that when the DN cell model was constructed, the IRF4 expression was significantly increased and that the related proteins of Notch1 and p-AKT (active state of AKT) in the downstream pathway of IRF4 were significantly upregulated. The extracellular matrix reconstruction-related protein collagen I was also significantly upregulated with the upregulation of IRF4. When IRF4 was knocked down in DN model cells, the expression levels of collagen I, Notch 1, and p-AKT were significantly decreased compared with those in the DN model group (Figures 3(a) and 3(b)). When the DN model was constructed in AB8/13 cells, the contents of inflammation- and immunity-related factors of IL-1*β* and IL-7 were significantly upregulated, and knocking down the IRF4 could almost reduce the factors to the normal control levels (Figure 3(c)).

### 3.2. Candidate Drug Screening for DN Treatment Based on IRF4

**Figure 4:**
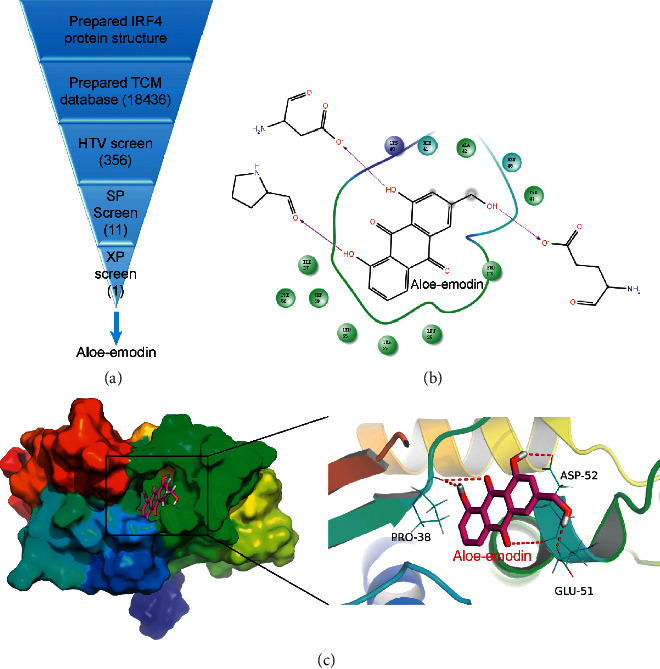
Receptor-ligand interactions of the compound. (a) Protocol flowchart of the IRF4 inhibitor screening strategy. Ae was screened through HTV, SP, and XP screening methods. (b) 2D binding model of the IRF4-Ae complex through the molecular docking method. (c) Effective binding of Ae to the active pocket of IRF4. Results showed that Ae interacted with three key amino acid residues of IRF4. TCM, traditional Chinese medicine; HTV, high-throughput virtual screening; SP, standard precision; XP, extra precision.

In accordance with the omics analyses and in vitro experiments results, IRF4 was identified to be an important gene in regulating the DN. Therefore, the IRF4 gene for drug screening was chosen. The systematic strategy for identifying TCM molecules was designed on the basis of the structures of IRF4 and TCM molecules. The HTV process is shown in Figure 4(a). The HTV screening method yielded 356 TCM molecules with the highest IRF4 score. Eleven TCM molecules were further screened from the previous 356 TCM molecules. Ae was screened to be the candidate target drug of the IRF4. Subsequently, the extra precision calculation was performed with the IRF4-Ae complex. Ae interacted with the key amino acid residues (i.e., Pro-38, Asp-52, and Glu-51) in the active pocket of IRF4 (Figures 4(b)–4(c)).

**Figure 5:**
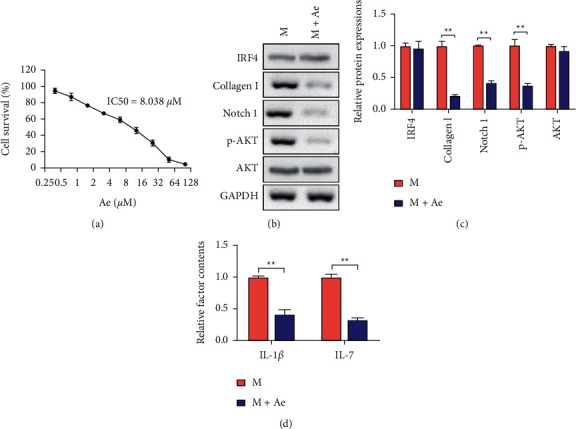
Amelioration of the DN in cell DN models by Ae. (a) Cell survival of DN model AB8/13 cells treated with different doses of Ae for 48 h IC50 = 8.038 *μ*M. (b) Representative bands of IRF4, collagen I Notch 1, p-AKT, AKT, and GAPDH proteins in the DN model and DN model + Ae-treated AB8/13 cells examined using Western blot analysis. (c) Comparison of the gray values of IRF4, collagen I, Notch 1, p-AKT, and AKT proteins. (d) ELISA results for the detection of IL-1*β* and IL-7 in cell medium between DN model and DN model + Ae-treated AB8/13 cells (mean ± s.d., *n* = 3 in triplicate, ^*∗∗*^*P* < 0.01).

The following in vitro experiments were designed to further analyze the effects of Ae on treating with DN. The effect of the Ae treatment for 48 h on cell viability was determined using the MTT assay, and results showed that the DN model AB8/13 cells were sensitive to Ae. The IC_50_ value of Ae was 8.038 *μ*M, and the concentration of Ae on the DN model AB8/13 cells was 2 *μ*M (Figure 5(a)). Western blot results showed that the addition of Ae had no effect on the IRF4 expression, but the downstream pathway-related proteins and collagen I were significantly downregulated (Figures 5(b) and 5(c)). Similarly, the addition of Ae could significantly decrease the factor contents of IL-1*β* and IL-7 in DN model cells (Figure 5(d)).

### 3.3. Ae Ameliorates the DN by Targeting IRF4 in a Rat DN Model

**Figure 6:**
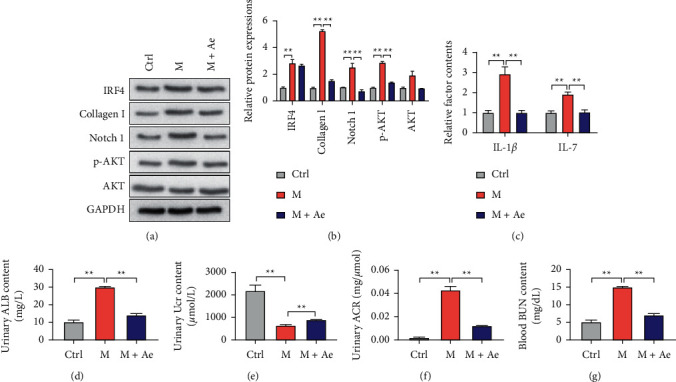
Amelioration of the DN by Ae through the targeting of IRF4 in DN model rats. (a) Representative bands of IRF4, collagen I, Notch 1, p-AKT, AKT, and GAPDH proteins examined using the western blot analysis of kidney samples from rats from the control, DN model, and DN model + Ae groups. (b) Comparison of the gray values of IRF4, collagen I, Notch 1, p-AKT, and AKT proteins. (c) ELISA results for the detection of IL-1*β* and IL-7 in serum samples from control, DN model, and DN model + Ae rats. Levels of (d) urinary ALB, (e) urinary Ucr, (f) urinary ACR, and (g) blood BUN in rats at eight weeks after the Ae treatment. ALB, urinary albumin; Ucr, urinary creatinine; ACR, ALB level/Ucr level; BUN, blood urea nitrogen (mean ± s.d., *n* = 6, ^*∗∗*^*P* < 0.01).

The effects of Ae on the DN progression in rats were examined. Kidney tissues were subjected to Western blot analysis, and results showed that the expression levels of IRF4, collagen I, Notch 1, and p-AKT were significantly upregulated when the DN model was constructed in rats. The oral treatment of Ae had no effect on the expression of IRF4, but the expression levels of collagen I, Notch 1, and p-AKT were significantly downregulated compared with those in the model group (Figures 6(a) and 6(b)). The serum samples were used for the detection of IL-1*β* and IL-7. ELISA results showed that the construction of the DN model could significantly increase the contents of these two factors, whereas the oral treatment of Ae significantly decreased the contents of IL-1*β* and IL-7 compared with those in the model group (Figure 6(c)). Subsequently, the urine samples were used for detecting the contents of ALB and Ucr. Results showed that the construction of the DN model significantly increased the content of ALB in rat urine, whereas the Ae treatment significantly relieved the increase in ALB in the DN model rats (Figure 6(d)). However, the content of Ucr in urine in the DN model group was significantly decreased compared with that in the control group, and the oral Ae treatment significantly increased the content of Ucr in model rats (Figure 6(c)). The trend of urinary ACR was consistent with the urinary ALB (Figure 6(f)). Lastly, the blood BUN was detected, and results showed that the content of blood BUN in the DN model group was significantly increased compared with that in the control group. The treatment of Ae could improve the BUN content (Figure 6(g), Supplemental Table 1).

## 4. Discussion

DN is one of the most important microvascular complications of diabetes and the primary cause of end-stage renal disease [23, 24]. In the course of DN, the morphology and the function of the glomeruli and its various intrinsic cells change. Among them, podocyte injury is closely related to proteinuria. The podocyte injury includes podocytic fusion, decreased podocyte number or density, podocyte apoptosis, podocyte epithelial-mesenchymal transdifferentiation, and podocyte hypertrophy [25–27]. These pathological changes are important factors for DN progression and key targets of drug therapy.

IRF4 is widely involved in the occurrence and the development of various diseases. Studies show that t(6; 14) (p25; q32) causes high IRF4 expression [28]. IRF4 can promote the transformation of fibroblasts and increase the apoptosis of lymphocytes with the IRF4 deletion, indicating the category of its oncogenes [29, 30]. Studies show that the main downstream regulatory factors of IRF4 are FK-BP3, MIG, FAIM, and ZFP94 [31]. IRF4 can inhibit apoptosis and promote proliferation. Like other oncogenes, activations of IRF4 and its downstream regulatory factors in tissues lead to the overexpression or the abnormal regulation caused by the ectopic or the heterotopic expression different from normal cells, which is an important reason for the occurrence of diseases. In this study, the bioinformatics analysis of omics data and in vitro and in vivo experiments reveal that during the occurrence of DN, the IRF4 expression is upregulated. At the same time, the IRF4 downstream pathways of Notch1 and p-AKT pathways are opened [32, 33], indicating that diabetes is stimulated by various factors, and IRF4 may promote renal damage. Further research results show that knocking down the TRF4 can reduce the disease progression of the DN. The low IRF4 level reduces the expression levels of collagen I, Notch1, and p-AKT.

In this study, Ae can play a protective role in multiple ways on the DN podocyte injury. The STZ-induced DN rat model is established. Compared with those in the model group, the expression levels of total IL-1*β*, IL-7, urinary ACR, and blood BUN in the Ae group are significantly reduced. These results indicate that the Ae has a protective effect on kidney damage in DN rats. The effects of Ae on IRF4, collagen I, Notch1, and p-AKT in renal tissues are observed. Western blot results show that Ae can significantly inhibit the expression levels of Notch1 and p-AKT proteins in the renal tissue induced by DN to reconstruct the extracellular matrix of the glomeruli. Ae can inhibit high-glucose-induced podocyte damage by regulating the downstream signaling pathways of IRF4, thereby inhibiting the renal interstitial fibrosis and inflammation. In which, the activities of IRF4 to the downstream pathways are probably inhibited through the target effects of Ae to the active pocket of IRF4. Thus, a low activity of IRF4 by Ae can equally reduce the expressions of collagen I, Notch1, and p-AKT, which subsequently improves DN.

The thickening of the basement membrane, increased number of renal interstitial tubules, and glomerular hypertrophy are the main pathological features in the early stage of DN. Considering the further development of the course of the disease, the accumulation of extracellular matrix, atrophy and disappearance of renal interstitial tubules, glomerulosclerosis, other late characteristics have appeared gradually, and renal tubular interstitial fibrosis has occurred. Recent studies show that renal tubulointerstitial fibrosis is the main pathologic basis and the common pathway of DN progression to end-stage renal failure. During DN progression, the expression of collagen type I is significantly increased and can be used as evidence for the occurrence of renal tubular interstitial fibrosis. In this study, compared with the model group, the Ae group has significantly decreased expression of collagen type I. Results show that Ae can play a protective role by inhibiting the process of renal tubulointerstitial fibrosis.

## 5. Conclusion

In summary, IRF4 is upregulated in the pathogenesis of DN. The IRF4 knockdown can reduce the podocyte damage in the DN. In addition, Ae can effectively improve the symptoms of DN and reduce the damage to renal tissue. The mechanism of action may be related to the inhibition of IRF4, Notch1, and p-AKT signaling pathways and the reduction of the collagen activity in renal tissue, thereby slowing down the progression of the DN. However, the functional characteristics and the advantages of Ae and the inhibitory mechanism of IRF4 remain to be further studied to provide a scientific basis and important support for new drug development and effective treatment of DN.

## Data Availability

The datasets used and/or analyzed are available from the corresponding author upon reasonable request.

## Supplementary Materials

Supplementary Materials Detection results of urinary albumin (ALB), urinary creatinine (Ucr), ALB level/Ucr (ACR), and blood urea nitrogen (BUN) in rats at eight weeks after the Ae treatment.
