# Supplementary material for: Aloe-Emodin Ameliorates Diabetic Nephropathy by Targeting Interferon Regulatory Factor 4
Source: Evid Based Complement Alternat Med. 2022 Apr 26;2022:2421624. doi: 10.1155/2022/2421624 (PMC9064522; doi:10.1155/2022/2421624)
Supplement: Supplementary Materials — Detection results of urinary albumin (ALB), urinary creatinine (Ucr), ALB level/Ucr (ACR), and blood urea nitrogen (BUN) in rats at eight weeks after the Ae treatment. [file 2421624.f1.pdf]

ALB (mg/L)	Ctrl	M	M + Ae	Week 8
	3.64	26.22	11.71	
	3.66	31.32	10.53	
	4.98	25.19	10.49	

Ucr (µmol/L)	Ctrl	M	M + Ae
	2467.52	660.93	909.10
	2003.11	676.38	910.00
	2070.89	591.67	881.02

urinary albumin to creatinine ratio (ACR) (mg/µmol)	Ctrl	M	M + Ae
	0.00	0.04	0.01
	0.00	0.05	0.01
	0.00	0.04	0.01
	3.64	26.22	11.71
	3.66	31.32	10.53
	4.98	25.19	10.49
	2467.52	660.93	909.10
	2003.11	676.38	910.00
	2070.89	591.67	881.02

BUN (mg/dL	Ctrl	M	M + Ae
	5.35	15.23	6.38
	5.44	14.28	7.40
	4.50	15.03	7.17

Ucr (mg/mL)	Ctrl	M	M + Ae
	37.91	77.64	41.37
	35.99	67.22	45.69
	33.00	72.86	42.33
